# The Effects of Continuous Usage of a Diabetes Management App on Glycemic Control in Real-world Clinical Practice: Retrospective Analysis

**DOI:** 10.2196/23227

**Published:** 2021-07-15

**Authors:** Yu-Zhen Tu, Ya-Ting Chang, Hung-Yi Chiou, Ken Lai

**Affiliations:** 1 H2 Inc Taipei Taiwan; 2 School of Public Health Taipei Medical University Taipei Taiwan

**Keywords:** app, diabetes care, diabetes, digital intervention, digital therapeutics, glycemic control, mobile app, mHealth, real-world data, therapy

## Abstract

**Background:**

The efficacy of digital technology in improving diabetes management has typically been demonstrated through studies such as randomized controlled trials, which have reported a steeper reduction in hemoglobin A_1c_ (HbA_1c_) values for patients who adopted a digital solution. However, evidence from real-world clinical practice is still limited.

**Objective:**

This study aimed to evaluate the effectiveness of digital interventions by tracking HbA_1c_ improvements over 1 year in real-world clinical settings.

**Methods:**

Patients used the Health2Sync mobile app to track self-measured outcomes and communicate with health care professionals (HCPs). HCPs used the web-based Patient Management Platform to monitor patient data, view test results from clinical laboratories, and communicate with patients. Patients who have been onboarded for at least 13 months and have consecutive HbA_1c_ findings for 5 quarters were included in the analysis. They were then stratified into 3 groups (high, mid, and low retention) based on their level of use of Health2Sync in the first 6 months of onboarding. A mixed model was built to compare the slopes of the rate of reduction in HbA_1c_ among the groups. In addition, these patients’ retention on the app from the seventh to the 12th month was verified through multiple comparisons.

**Results:**

A sample of 2036 users was included in the analysis. With the mixed model coefficient estimates, we found that app users had significant HbA_1c_ percentage reductions as the passed quarter count increased (*t*=–9.869; *P*<.001), and that effectiveness increased in the high (*t*=–5.173) and mid retention (*t*=–6.620) groups as the interaction effects were significantly negative compared to that in the low retention group (*P*<.001) in the passed quarter count. The low retention group also had the highest average HbA_1c_ value at the end of 13 months (high: 7.01%, SD 1.02%; mid: 6.99%, SD 1.00%; low: 7.17%, SD 1.14%) (Bonferroni correction: high vs low, *P*=.07; mid vs low, *P*=.02; high vs mid, *P*>.99). The level of use of the app remained consistent in the seventh to the 12th month after onboarding (high: 5.23 [SD 1.37] months, mid: 2.43 [SD 1.68] months, low: 0.41 [SD 0.97] months) (*P*<.001).

**Conclusions:**

Our analysis shows that continuous usage of the diabetes management app is associated with better glycemic control in real-world clinical practice. Further studies are required to reveal the efficacy for specific diabetes types and to observe effects beyond 1 year.

## Introduction

Global diabetes prevalence in the adult population is estimated to have grown from 8.5% in 2014 to 9.3% in 2019 and is projected to be over 10% by 2030 [1,2]. In Taiwan, the prevalence of diabetes approached 9.8% in 2016 [3]. Caring for patients with diabetes and a couple of its associated complications—chronic kidney disease and acute kidney failure—costed more than US $2.5 billion in Taiwan in 2019, which accounted for 11.4% of the total health care expenditure in that year [4]. As the cost of diabetes care continues to increase in the foreseeable future, health care providers will need to consider adopting digital solutions to manage patients with diabetes in an effective and scalable manner.

Increasing evidence suggests that interventions with digital technology enhance the effect of conventional care practices on patients with diabetes. For example, patients who received coaching and decision support from the WellDoc app presented greater reductions in hemoglobin A_1c_ (HbA_1c_) levels compared to those in the control group [5,6]. A cohort study using the FareWell app suggested the same effect on adults with type 2 diabetes, and the users reported greater confidence in managing the disorder [7]. Furthermore, multiple meta-analyses on randomized controlled trials (RCTs) have shown that interventions including mobile apps help patients, especially those with type 2 diabetes, to lower their HbA_1c_ levels significantly without notable adverse effects [8-14]. These apps usually allow patients with diabetes to log and visualize their self-monitored data, provide education and feedback, or provide communication channels between patients and their caregivers or peers [12,13,15]. From the patient’s perspective, these features were also deemed as characteristics of a great app [16].

Although the aforementioned mobile apps are intended to be used by patients in their daily lives, few studies have verified the effects of mobile apps with real-world data. A previous study [17] using the mobile version of the One Drop diabetes management platform reported a substantial reduction in HbA_1c_ values of users for the first 2 entries in the app regardless of diabetes type, but the study did not compare effects between app users and nonusers and did not examine the long-term effects of app engagement [17].

This study aims to address the aforementioned issues through a retrospective analysis. We evaluated the difference in HbA_1c_ improvement between active app users and those who drop out from the app in a real-world context, and monitored their glycemic control status for 1 year.

## Methods

### Health2Sync App and Patient Management Platform

The Health2Sync mobile app and web-based Patient Management Platform were launched in 2014 to support patients with chronic diseases such as diabetes. The Health2Sync solution was developed to support patients to make behavioral changes through a do-track-learn cycle and enable those around the patient to care for the patient. The mobile app, intended for patients, is free to download for iOS and Android users worldwide. The Patient Management Platform is designed for health care professionals (HCPs) to care for patients in clinics or hospitals remotely.

**Figure 1:**
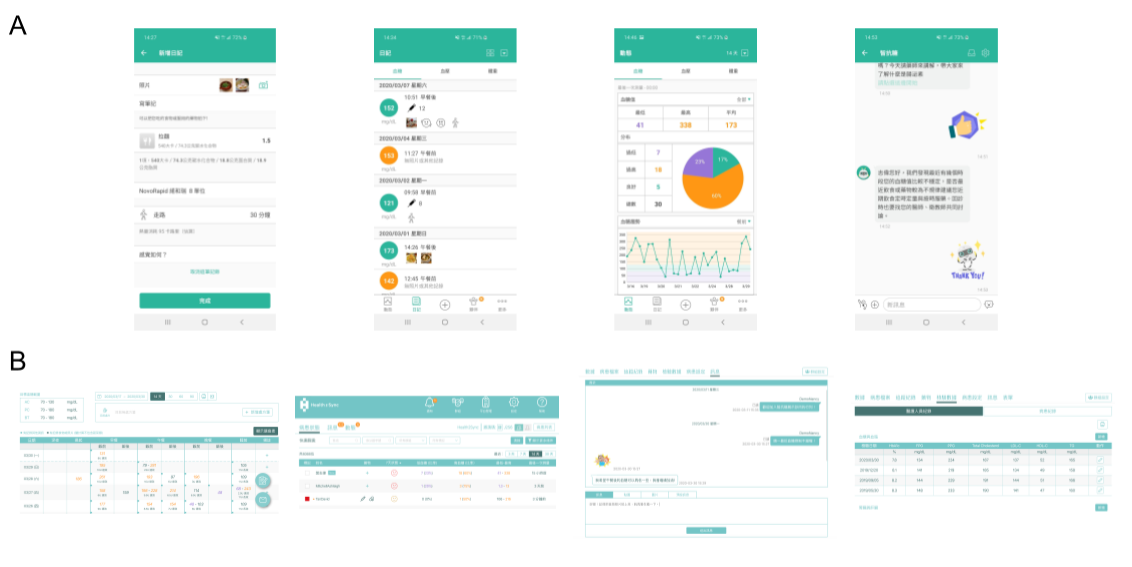
Screenshots of the Health2Sync app and Patient Management Platform.

The Health2Sync app has numerous features that support diabetes management (Figure 1A). To start with, users can record their self-measured outcomes manually or by synchronizing with glucose meters, sphygmomanometers, and weight scales. They can also log their daily behaviors such as diet, medication, and exercise. Users can review their past records to remind themselves of how their behaviors are linked to the outcomes. On the dashboard, users can visualize their recent progress and trends by referring to simple charts and statistics. Health2Sync’s most differentiating feature is its “Partners” function, which enables users to connect with HCPs, family members, or peers so that they can collaborate with the patient to manage his/her condition. The “Partners” feature allows hospitals or clinics that use the Patient Management Platform to view the patient’s data and communicate with the patient through a messaging feature on the platform. Every user of the app also has the Health2Sync bot as a default partner that provides automated analyses, alerts, encouragements, and educational content. In addition, the app has a peer functionality that lets users learn and interact with other patients with diabetes who use the app.

The recorded outcomes and behavioral data from patients will be sent in real time to the Patient Management Platform to be reviewed by HCPs (Figure 1B). On the platform, HCPs can also send messages to patients to provide care and answer questions. From the Patient Management Platform’s dashboard, HCPs can quickly scan their pool of patients to check their latest status and identify any patients requiring immediate care. The alert functionality will notify HCPs of critical patient trends or events. The Patient Management Platform can also be integrated with clinical laboratories so that HCPs can view the results of laboratory tests directly on the platform. Test results including those for HbA_1c_, blood glucose, lipid profile, and renal and liver function are supported.

### Data Collection

Users’ HbA_1c_ records on the Patient Management Platform were included. All the HbA_1c_ values were either inputted by HCPs or synchronized from clinical laboratories. To assess the effects of the intervention at different time points, we looked at when each patient added a clinic or hospital as a partner in Health2Sync and determined how many months after adding the partner the HbA_1c_ values were recorded. The calculation method considered the difference in the number of days between the date when users added the partner and that of each HbA_1c_ recording, divided by 30. According to the calculated numbers, these records were then categorized into five time buckets from B_0_ to B_4_: [–2, 1), [1, 4), [4, 7), [7, 10), and [10, 13). The buckets were designed to reflect the common practice of determining HbA_1c_ levels once every 3 months in Taiwan, and that the first HbA_1c_ record can be in –2~1 month from the time when the partnership was created. We believe that as glycated hemoglobin levels typically reflect blood glucose values in the past 8-12 weeks [18], HbA_1c_ levels in the first month of the partnership would only be minimally affected by the intervention.

### Subjects

**Figure 2:**
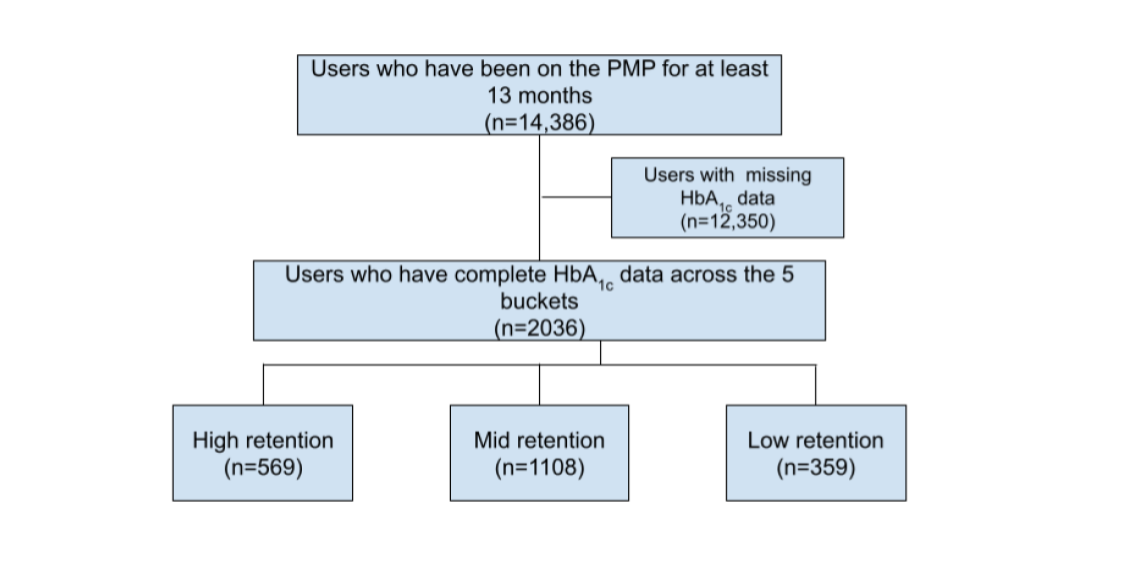
Inclusion flow chart of this study. The final 3 groups were separated by their retention in the first 6 months. Sample sizes of each stage are noted. PMP: Patient Management Platform.

Health2Sync users who were connected to clinics or hospitals via the Health2Sync platform for at least 13 months were included, thus yielding an initial pool of 14,386 users. Those with missing data in any of the 5-bucket HbA_1c_ observation periods were excluded owing to the requirement of a complete data set for subsequent modeling. At this stage, we excluded 12,350 users and retained 2036 users. These users were then separated into 3 groups in accordance with their app retention. In the first 6 months after adding the partner, users who opened the Health2Sync app every month were categorized as the “high retention” group, those who opened the app in only the first month were categorized as the “low retention” group, and the remaining patients were categorized as the “mid retention” group. Eventually, there were 569, 1108, and 359 users in the high, mid, and low retention groups, respectively. Demographic data of the included 2036 users and their distribution across the 3 retention groups are depicted in Table 1, although the majority of these included users preferred not to disclose their demographic status. Throughout this study, all users continued to receive diabetes care from the same clinic or hospital, otherwise there would be no HbA_1c_ records on the Patient Management Platform. Figure 2 shows the inclusion flow chart described above.

### Analyses

All the analyses in this study were performed with R (version 3.6.1, The R Foundation) [19].

#### User Characteristics

All users filled an onboarding form in the Health2Sync app where they self-reported their age, gender, and diabetes type. One-way analysis of variance (ANOVA) and the Pearson chi-square test were used for continuous and categorical variables, respectively, to assess the homogeneity of demographics across the 3 retention groups.

#### HbA_1c_ Changes

Mean (SD) HbA_1c_ levels of each group in B_0_ and B_4_ were calculated to compare the glycemic control status by app retention levels. One-way ANOVA was used to examine the heterogeneity across groups, and the pairwise *t* test with Bonferroni correction was applied for post hoc analysis.

We used the rate of change in HbA_1c_ levels, generated using the formula (HbA_1c_ value - mean HbA_1c_ in B_0_) / mean HbA_1c_ in B_0_, to assess the improvement in glycemic status across the groups. The rate of change was used instead of the absolute magnitude of change because users who had higher HbA_1c_ levels in B_0_ also had more room for a reduction in its value; hence, the comparison of changes in absolute HbA_1c_ values across groups would be biased. In addition, considering that the reduction in HbA_1c_ levels was nonlinear with time, all the changes in rates were added by a constant so that the minimum value is 1, and all these values were log-transformed. These log-transformed values were incorporated in the mixed model as the dependent variable, where fixed effects included the time bucket numbers (ie, quarters passed, from 0 to 4), app retention groups, and their interaction. Each user’s intercept was controlled in the random effects model [20,21].

#### Monitoring of App Retention

To follow the users’ app retention in the subsequent 6 months (ie, months 7-12 after partnership creation), we enumerated the months after which they had opened the app. The purpose of this analysis is to verify whether these 3 groups of users maintained their usage behavior in the following months of the retrospective observation period. The differences across these groups were assessed using 1-way ANOVA, and a pairwise *t* test with Bonferroni correction was used for post hoc analysis.

## Results

Table 1 presents user characteristics stratified by the 3 user groups based on different levels of app retention. Users were not required to provide their demographic profiles; hence, sample sizes may differ across demographic characteristics. We found no significant difference in age (*F*_2,806_=1.441; *P*=.24) and gender (*χ*^2^_22_=0.3637; *P*=.83) distributions across the 3 groups; however, the diabetes type distribution was not even in these groups (*χ*^2^_24_=27.489; *P*<.001).

**Table 1:** User characteristics stratified by app retention (N=2036).

Characteristics		Total	High retention (n=569)	Mid retention (n=1108)	Low retention (n=359)	*P* value	
Age (years), mean (SD); n		57.7 (13.4); 809	57.1 (12.3); 297	58.7 (13.7); 327	57.1 (14.4); 185	.24	
**Gender, n**							.83
	Male	506	226	175	105		
	Female	461	214	157	90		
**Diabetes type, n**							<.001
	Type 1	53	21	10	22		
	Type 2	859	384	311	164		
	Others	33	24	6	3		
HbA_1c_ in time bucket B_0_ (%), mean (SD)		7.90 (1.74)	7.99 (1.86)	7.92 (1.72)	7.70 (1.60)	.04	
HbA_1c_ in time bucket B_4_ (%), mean (SD)		7.03 (1.03)	7.01 (1.02)	6.99 (1.00)	7.17 (1.14)	.02	

**Figure 3:**
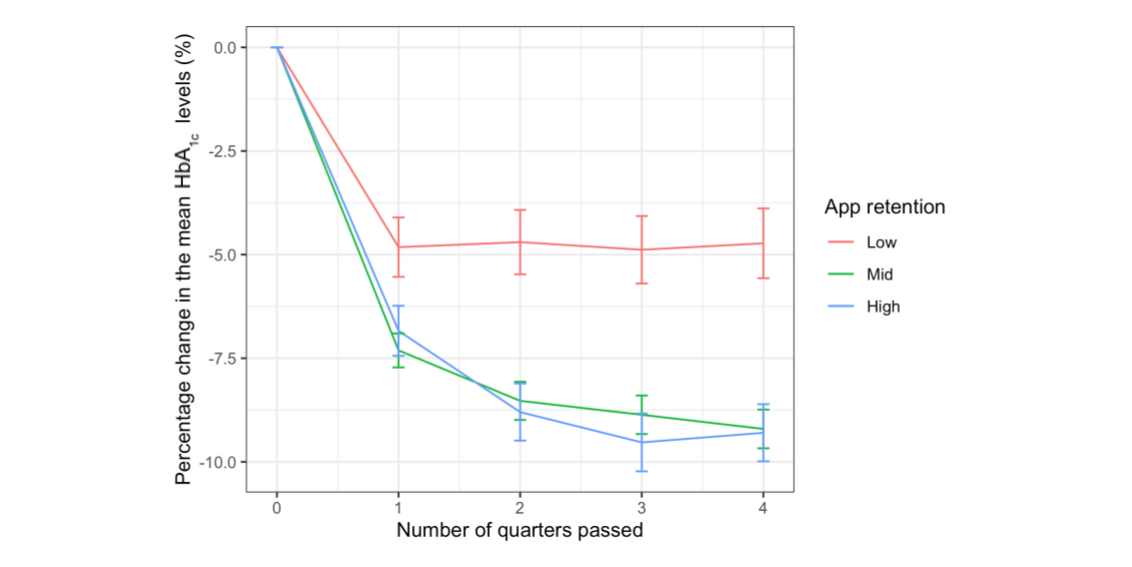
Users' averaged percentage change in HbA_1c_ levels in each time bucket. The error bars represent the SE among users in the group.

**Figure 4:**
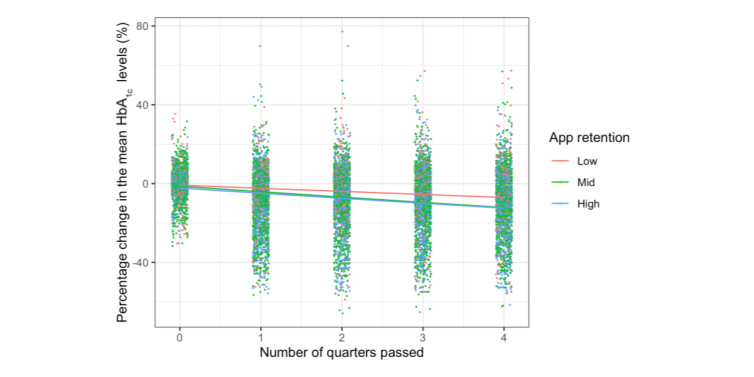
Jittered scatter plot depicting the relationship between HbA_1c_ percentage changes and time periods after joining the Patient Management Platform. Each dot represents 1 user's data at the time. The overlaid regression lines are based on the estimated coefficients from the mixed model.

Users with higher app retention presented a greater reduction in the rate of change in HbA_1c_ levels (Figure 3). The group with the lowest retention presented the smallest reduction in HbA_1c_ levels in time bucket B_1_ (–4.8%, SD 13.6%) and remained constant afterward up to time bucket B_4_ (–4.7%, SD 15.9%), while the other groups presented a steeper reduction in time bucket B_1_ (mid: –7.3%, SD 13.6%; high: –6.8%, SD 14.4%), and the decreasing trends continued up to time bucket B_4_ (mid: –9.2%, 15.5%; high: –9.3%, SD 16.4%). The mixed model further verified the significance of this effect (Figure 4). Users presented significant HbA_1c_ percentage reductions as the passed quarter count increased (*β* estimate=–9.626×10^-3^; *t*=–9.869; *P*<.001). Furthermore, being in mid and high retention groups further augmented this reduction with time as the interactions were significantly negative when the low retention group was set as the baseline for the past quarter (mid: *β* estimate=–7.360×10^-3^; *t*=–6.620; *P*<.001; high: *β* estimate=–6.419×10^-3^; *t*=–5.173; *P*<.001). When the high retention group was set as the baseline for comparison, no significant difference was found between the augmentation effects between the mid and high retention groups for the past quarter (*β* estimate=–9.410×10^-4^; *t*=–1.007; *P*=.31). No main effect was observed for the retention groups when the low retention group was set as the baseline for comparison (mid: *β* estimate=–3.294×10^-3^; *t*=–0.691; *P*=.49; high: *β* estimate=–8.951×10^-3^; *t*=–1.687; *P*=.09).

**Figure 5:**
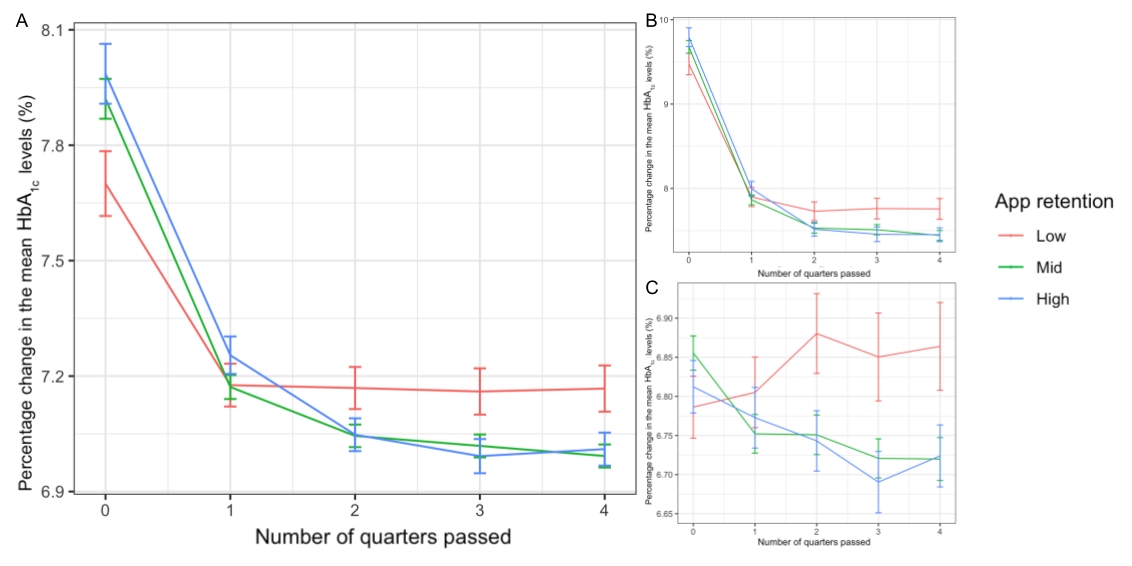
Users' mean HbA_1c_ in each time bucket. The error bars represent the standard errors among users in the group.

The high and mid retention groups also presented a greater reduction in raw HbA_1c_ values and lower HbA_1c_ levels at the end of the observation period, although their raw HbA_1c_ values were higher in time bucket B_0_ (Table 1 and Figure 5A). We observed significant differences in HbA_1c_ levels between the high and low retention groups in time bucket B_0_ (high: 7.99%, SD 1.86%; 95% CI 7.83%-8.14%; mid: 7.92%, SD 1.72%; 95% CI 7.82%-8.02%; low: 7.70%, SD 1.60%; 95% CI 7.54%-7.87%) (Bonferroni correction: high vs low, *P*=.045; mid vs low, *P*=.11; high vs mid, *P*>.99) and between mid and low retention groups in time bucket B_4_ (high: 7.01%, SD 1.02%; 95% CI 6.93%-7.09%; mid: 6.99%, SD 1.00%; 95% CI 6.93%-7.05%; low: 7.17%, SD 1.14%; 95% CI 7.05%-7.29%) (Bonferroni correction: high vs low, *P*=.07; mid vs low, *P*=.02; high vs mid, *P*>.99). In a stratified analysis, users with HbA_1c_ values of ≥8% in time bucket B_0_ presented no significant difference in glycemic status at the beginning (high: 9.79%, SD 1.66%; 95% CI 9.58%-10.01%; mid: 9.68%, SD 1.54%; 95% CI 9.53%-9.83%; low: 9.48%, SD 1.41%; 95% CI 9.23%-9.73%) (*F*_2_=1.632; *P*=.20), but the difference in HbA_1c_ levels between the mid and low retention groups in time bucket B_4_ was still significant (high: 7.45%, SD 1.23%; 95% CI 7.29%-7.61%; mid: 7.44%, SD 1.21%; 95% CI 7.33%-7.56%; low: 7.76%, SD 1.36%; 95% CI 7.52%-8.00%) (Bonferroni correction: high vs low, *P*=.08; mid vs low, *P*=.04; high vs mid, *P*>.99) (Figure 5B). Similar results were obtained among users with HbA_1c_ values of <8% in time bucket B_0_ (high: 6.81%, SD 0.62%; 95% CI 6.75%-6.88%; mid: 6.86%, SD 0.57%; 95% CI 6.81%-6.90%; low: 6.79%, SD 0.61%; 95% CI 6.71%-6.86%) (*F*_2,1269_=1.425; *P*=.24) and in time bucket B_4_ (high: 6.72%, SD 0.74%; 95% CI 6.65%-6.80%; mid: 6.72%, SD 0.72%; 95% CI 6.67%-6.77%; low: 6.86%, SD 0.86%; 95% CI 6.75%-6.97%) (Bonferroni correction: high vs low, *P*=.08; mid vs low, *P*=.03; high vs mid, *P*>.99) (Figure 5C).

**Figure 6:**
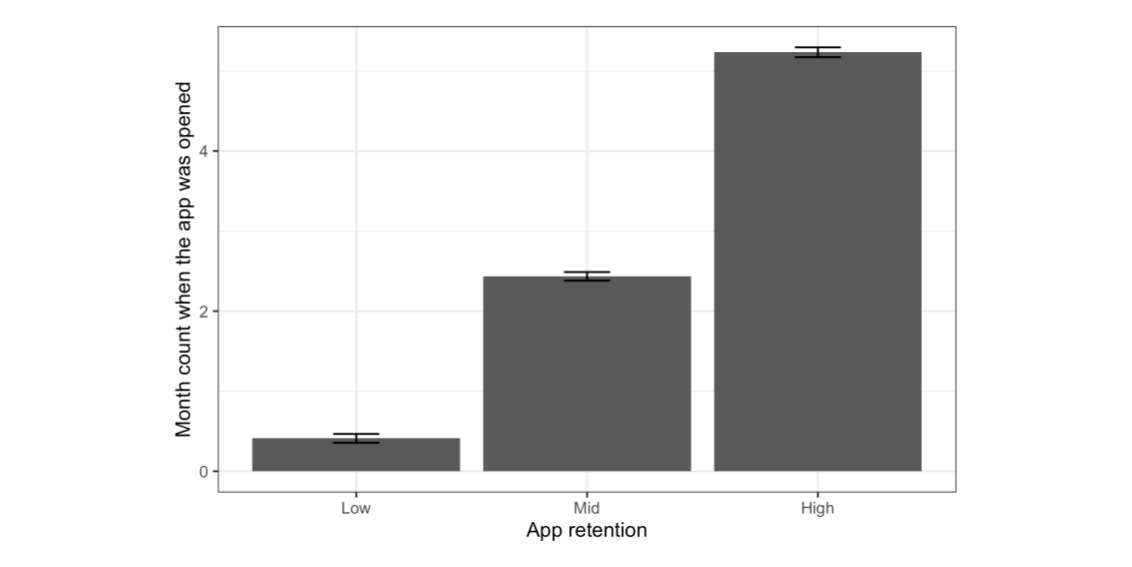
Month count when the app was opened during the seventh to 12th month. The heights of the bars and the ranges of error bars represent the mean (SE) values in each group, respectively.

Users’ retention behavior patterns remained the same in the second half of the tracking period (Figure 6). One-way ANOVA revealed significant differences in app retention across the 3 groups (*F*_2,1797_=1082; *P*<.001), and subsequent post hoc analyses using Bonferroni correction revealed significant differences among the groups (high: 5.23 [SD 1.37] months, mid: 2.43 [SD 1.68] months, low: 0.41 [SD 0.97] months) (*P*<.001) for all comparison pairs.

## Discussion

### Principal Findings

In this study, we found that patients with diabetes, who continuously used the Health2Sync app and were supported by HCPs who used the Health2Sync Patient Management Platform, presented a steeper reduction in HbA_1c_ levels and achieved lower HbA_1c_ levels after 1 year, compared to patients who dropped out and received traditional care for 1 year. Although we only used the app usage behavior data in the first 6 months to stratify the patients, their app usage patterns remained consistent in the following months. Additionally, our study shows positive results with regard to diabetes management from real-world data without prior RCT settings. While an RCT has an advantage over real-world evidence in providing homogeneous study groups for comparisons with designed inclusion and exclusion criteria, it is incapable of reflecting actual clinical practice where heterogeneous scenarios exist [22,23]. As digital interventions are to be applied to all patients, we believe that our study with real-world evidence is more convincing in demonstrating efficacy.

In our study, both the mid and high retention groups presented a greater reduction in HbA_1c_ levels than their counterparts in the low retention group. In other words, only users who stopped using the app in the first month after adding a clinic or hospital as a partner saw poorer HbA_1c_ improvements than others. The beneficial effects of the Health2Sync app and Patient Management Platform might result from the in-app structured display and the general education or personalized feedback that further enhance users’ health awareness, which were reported to be associated with better HbA_1c_ outcomes [13,24].

Although the majority of included subjects chose not to disclose their diabetes type, and the sample size was unbalanced across known diabetes types and retention groups, we performed a subgroup analysis to investigate the effect of app retention on different diabetes types, specifically type 1 and type 2. Based on changes in the raw HbA_1c_ values, we found that the reduction in the HbA_1c_ levels of only the patients with type 2 diabetes was related to app retention, while that of patients with type 1 diabetes seemed unrelated to app adherence (Multimedia Appendices 1 and 2 show the trend in app retention for patients with type 1 and type 2 diabetes, respectively). Many studies have focused on the efficacy of digital interventions for patients with type 2 diabetes or provided inadequate evidence of the efficacy of such interventions among patients with type 1 diabetes [7,9,14]. Some studies or analyses reported positive outcomes among patients with type 1 diabetics, but the effects were not as large as those among patients with type 2 diabetes [8,13,17].

### Limitations

Our data also suggest such differences between the patients with type 1 and those with type 2 diabetes. However, considering the limited sample, future studies are needed to investigate whether the benefits from digital tools can also be applied to patients with type 1 diabetes, especially in real-world settings. Another study limitation is that we did not consider the differences in medication and daily behaviors such as exercise across patients, and these factors could have vital impacts on glycemic control. Future studies should also include these features in analyses.

With the growing prevalence of diabetes, it may become increasingly difficult to care for patients without increasing the number of HCPs. As such, digital solutions can play a critical role to enhance and scale diabetes care [25]. Moreover, during the COVID-19 pandemic, diabetes was found to be a common comorbidity, and patients with diabetes were found to be more vulnerable to COVID-19 [26-28]. A mobile app and platform that can enable HCPs to practice remote care should be helpful for glycemic control and can reduce the risk of people with diabetes being infected during visits to clinics or hospitals [29].

### Conclusions

In conclusion, our retrospective analyses show that continuous usage of the Health2Sync app and Patient Management Platform was helpful for improving HbA_1c_ levels. Further studies are needed to reveal the efficacy of such interventions for specific diabetes types and to observe the effects beyond 1 year.

## Appendix

Multimedia Appendix 1 Diabetes type 1 users' mean HbA<sub>1c</sub> in each time bucket. The error bars represent the standard errors among users in the group.

Multimedia Appendix 2 Diabetes type 2 users' mean HbA<sub>1c</sub> in each time bucket. The error bars represent the standard errors among users in the group.

## Abbreviations

ANOVA: analysis of variance
HbA_1c_: hemoglobin A_1c_
HCP: health care professional
RCT: randomized controlled trial
