# Supplementary figures and images for: The Effects of Continuous Usage of a Diabetes Management App on Glycemic Control in Real-world Clinical Practice: Retrospective Analysis
Source: J Med Internet Res. 2021 Jul 15;23(7):e23227. doi: 10.2196/23227 (PMC8323018; doi:10.2196/23227)

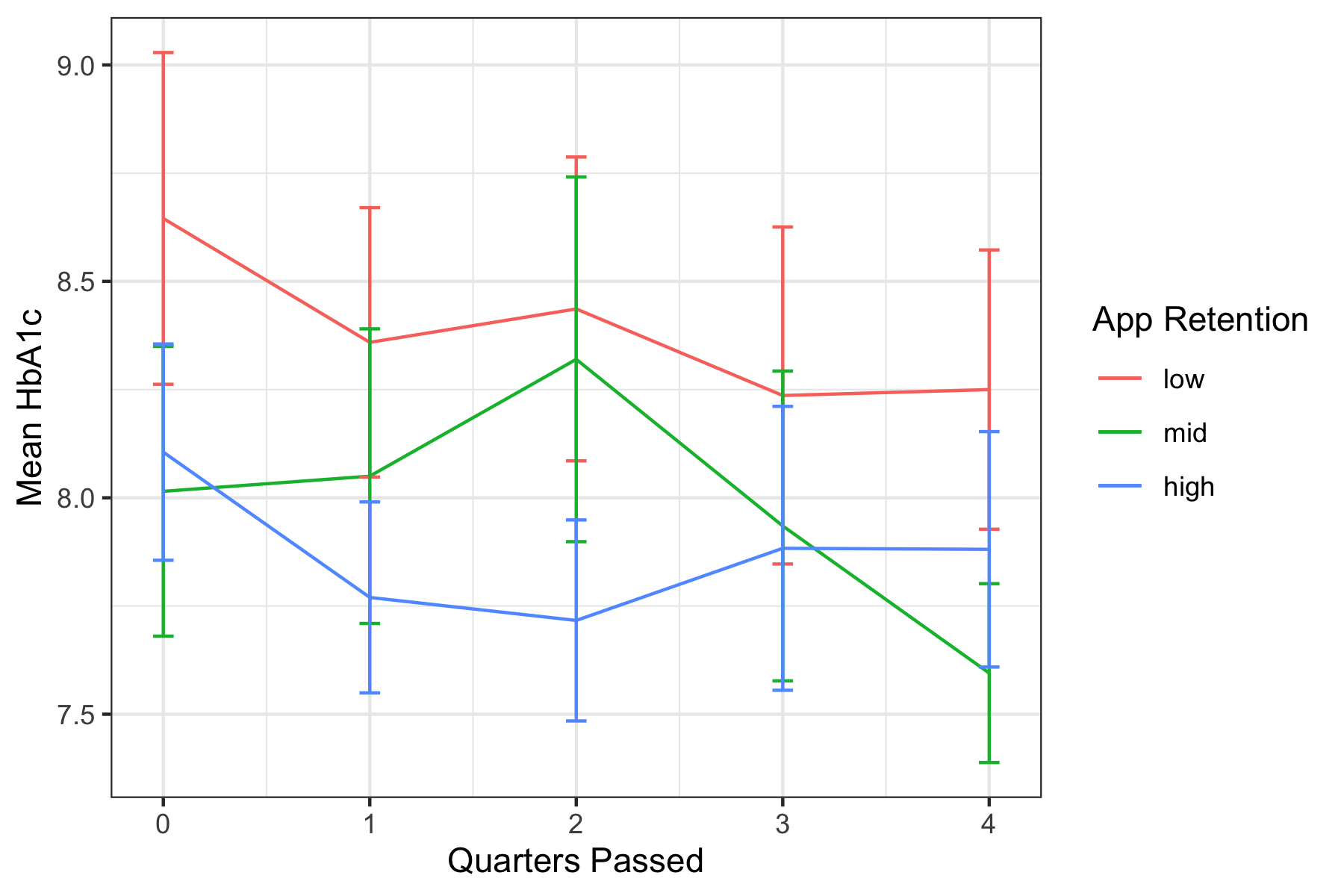

Supplement: Multimedia Appendix 1 [file jmir_v23i7e23227_app1.png]

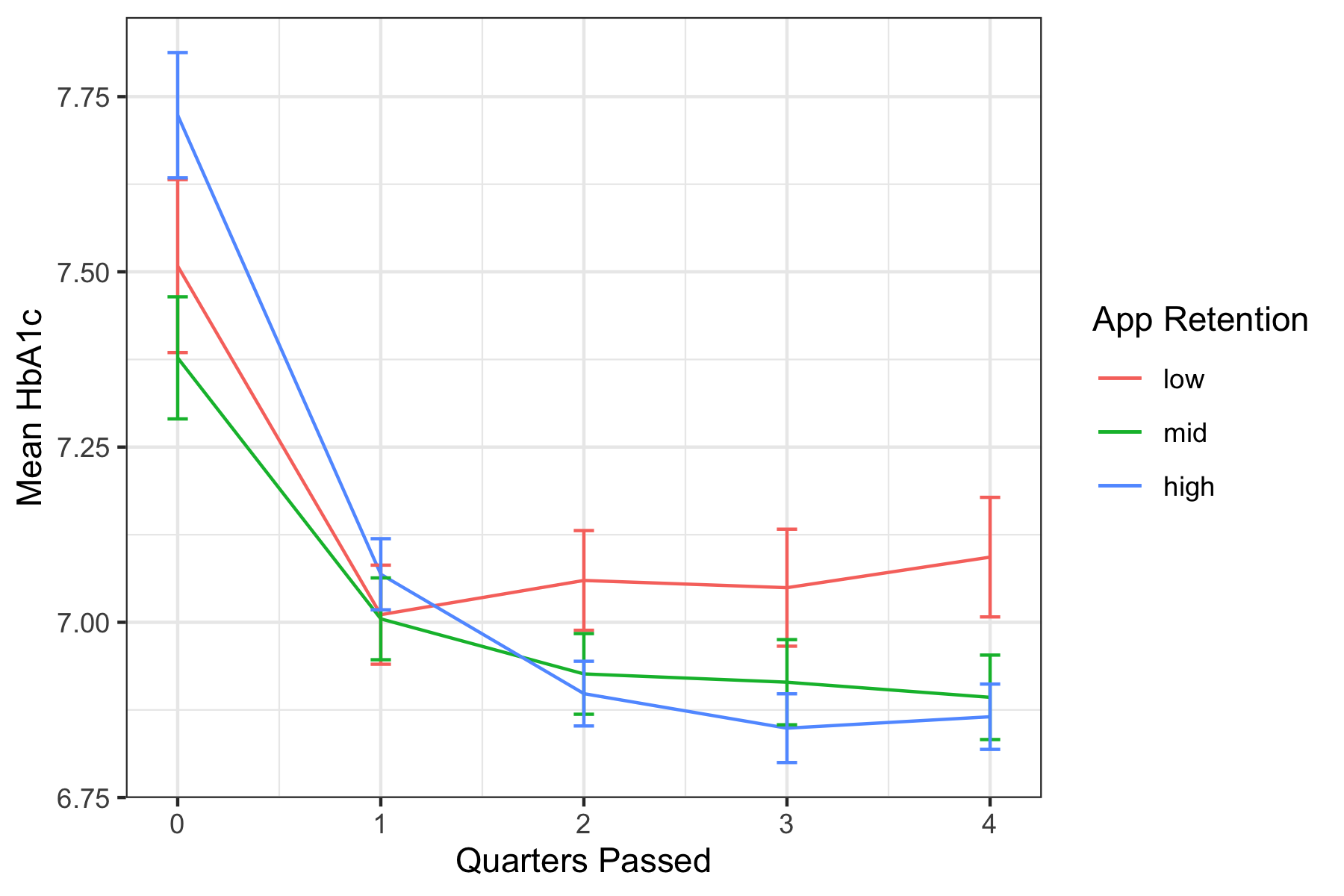

Supplement: Multimedia Appendix 2 [file jmir_v23i7e23227_app2.png]
